# Association Between Pad Placement and the Return of Spontaneous Circulation for Defibrillation During Ventricular Fibrillation: A Systematic Review

**DOI:** 10.19102/icrm.2021.120607

**Published:** 2021-06-15

**Authors:** David Paré, Julien Blais L’Écuyer, Éric Mercier

**Affiliations:** ^1^Département de médecine familiale et médecine d’urgence, Université Laval, Québec, Québec, Canada; ^2^VITAM – Centre de recherche en santé durable de l’Université Laval, Québec, Québec, Canada

**Keywords:** Cardiac arrest, defibrillation, pad placement, return of spontaneous circulation, ventricular fibrillation

The importance of early defibrillation in the management of ventricular fibrillation (VF) or pulseless tachycardia (pVT) is well-established. Prompt defibrillation and first shock success have been linked to an increased return of spontaneous circulation (ROSC) and higher survival rates.^1^ The American Heart Association (AHA) and European Resuscitation Council guidelines on cardiac arrest management recommend placement of defibrillation pads in the conventional sternal–apical or anterolateral position during defibrillation, and other potentially acceptable positions such as anteroposterior, bi-axillary (each side of the chest wall), left precordium–left scapula, and apical electrode–right scapula have also been described.^2^

We conducted a systematic review aiming to determine whether pad placement influences ROSC or survival rates in adult patients with VF or pVT requiring urgent external defibrillation. The study protocol was registered beforehand in the International Prospective Register of Systematic Reviews (registration no. CRD42018103400). A search strategy was developed for three databases (Medline/PubMed, Embase, and the Cochrane Central Register of Controlled Trials), last updated on May 18, 2020. The grey literature and clinical trial registries were also scrutinized for additional relevant studies. We considered for inclusion only randomized controlled trials designed to compare the effectiveness of different pad placements to facilitate ROSC and/or survival in adults requiring urgent external defibrillation for VF or pVT. The primary objective was to determine the association between pad placement and ROSC. Secondary outcomes assessed were survival, survival with good neurological outcomes, and the average number of electrical shocks required to achieve ROSC. A risk-of-bias assessment was completed using the Cochrane Collaboration Tool for observational studies and randomized controlled trials.

A total of 822 unique citations were retrieved by our search strategy. However, only one study fulfilled our inclusion criteria.^3^ The included study compared biphasic and monophasic waveforms for transthoracic defibrillation of VF during routine electrophysiological testing. Measurements were performed during the insertion of an implantable cardioverter-defibrillator and at hospital discharge. They used a binary step-up protocol: if the implantable cardioverter-defibrillator failed to defibrillate an episode, patients were randomized to receive a back-up shock either monophasic or biphasic in nature, from an external defibrillator. Electrode pad positions were changed after the initial seven patients and crossover was performed between the sessions to evaluate the secondary outcome of pad positioning [anteroposterior (AP) vs. anterolateral (AL)] on the efficacy of external defibrillation after failed internal shocks. Among the 50 episodes (n = 14 patients) of VF recorded, no statistical difference was recorded between AP and AL pad placement in achieving ROSC relative to the first attempt (88% vs. 68%; odds ratio: 3.45, 95% confidence interval: 0.79–15.01; p = 0.0987). A statistically significant difference was observed when monophasic waveforms were used as 83% (10/12) of VF episodes were successfully converted to ROSC using the AP position, while only 36% (4/11) were converted using the AL position (36%) (p < 0.05). This finding was not replicated when the biphasic waveform was used. The overall risk of bias was considered to be of a moderate degree.

Our systematic review therefore highlights the important and persistent knowledge gap relative to electrode pad placement for defibrillation of VF or pVT during adult cardiac arrest. The only included study was a controlled trial of 14 patients conducted in an electrophysiology setting. Current recommendations are therefore based almost exclusively upon expert opinion and animal model studies.

The impact of electrode pad placement during cardioversion on the successful restoration of sinus rhythm in patients with atrial fibrillation (AF) has been studied more extensively. A systematic review that included 13 studies showed that electrode pad placement might not be a critically important factor in successful cardioversion of AF.^4^ Furthermore, a recent randomized controlled trial published in 2020 observed no difference between AL and AP regarding the success rate in restoring normal sinus rhythm in the emergency department among patients with recent-onset AF.^5^ During cardiac arrest, early access to defibrillation is known to improve survival rates, in particular with good neurological outcomes. However, while more literature is emerging on advanced interventions such as dual sequential defibrillation or even extracorporeal membrane oxygenation (ECMO) in patients with refractory VF, only low-quality evidence is available at this time for defibrillation relative to the impact of pad placement.

A human experimental study on transthoracic defibrillation found no difference between pad position on transthoracic impedance (TTI), which is postulated to affect defibrillation success rates.^6^ One experimental study confirmed an advantage when using electrode pads in the AP position for reducing myocardial damage.^7^ Concomitantly, in the latest study, positioning the second electrode on the mid-axillary line was associated with a lower risk of myocardial injury than AP or AL while also reducing the risk for myocardial damage. In our included study,^3^ the position of the electrode pads—either AP or AL—had no influence on the biphasic shock efficacy to achieve ROSC, but the AP pad position was more effective when conducting monophasic shocks. One prospective study of 86 healthy volunteers compared two conventional pad placements—AL (subclavicular/subaxillar) and AP—to measure TTI.^8^ Their results suggested that the AP position is favorable for defibrillation from a theoretical perspective. However, another similar study that compared TTI using three AHA-recommended pad positions (anterior–apex, apex–posterior, and AP) reported recorded TTIs for all three positions.^9^ An animal model study using swine reported that minor variations in pad placement can impact the defibrillation success rate.^10^ Considering animal model and healthy volunteer studies, there is a high likelihood that pad placement might influence defibrillation success rates. However, the applicability and translation of the findings obtained during animal model or healthy volunteer studies to the context of nonexperimental adult cardiac arrest remain unknown.

Our systematic review underlines the limited evidence that currently exists regarding the basics of external defibrillation, such as the impact of different pad placements on patient-critical outcomes. We believe that, despite the recent enthusiasm for many new technologies, the conduct of studies designed to optimize interventions with a proven value, such as pad placement during defibrillation, remains a key objective to improve the prognosis of patients following cardiac arrest.
